# Dietary supplementation with fermented rapeseed and seaweed modulates parasite infections and gut microbiota in outdoor pigs

**DOI:** 10.3389/fvets.2025.1565686

**Published:** 2025-06-19

**Authors:** Charlotte Smith Bonde, Frida Brasen Drøhse, Nilay Büdeyri Gökgöz, Lukasz Krych, Dennis Sandris Nielsen, Heidi Huus Petersen, Rikke Matthiesen, Ninfa Rangel Pedersen, Peter Geldhof, Andrew R. Williams, Stig Milan Thamsborg, Helena Mejer

**Affiliations:** ^1^Department of Veterinary and Animal Sciences, University of Copenhagen, Frederiksberg, Denmark; ^2^Department of Food Science, University of Copenhagen, Frederiksberg, Denmark; ^3^Danish Veterinary and Food Administration, Glostrup, Denmark; ^4^Fermentationexperts A/S, Copenhagen, Denmark; ^5^Laboratory of Parasitology, Faculty of Veterinary Medicine, Ghent University, Ghent, Belgium

**Keywords:** parasite, bioactive forage, gut microbiome, brown seaweed, macroalgae, rapeseed meal, *Ascaris suum*, swine

## Abstract

Outdoor pig production systems can increase exposure to helminth infections such as *Ascaris suum*. Anthelmintic drug treatments are currently the primary strategy for controlling such infections; however, this approach is considered unsustainable due to the risk of parasites developing anthelmintic resistance. This study investigated the potential anthelmintic effect of a 2% w/w fermented rapeseed–seaweed (FRS) dietary supplement administered over an 11–12 week period in growing-finisher pigs. Outcomes assessed included parasitic fecal egg counts (FEC), serology, gut microbiota composition, and systemic inflammation. The FRS supplement consisted of 6% *Saccharina latissima*, 6% *Ascophyllum nodosum*, and 88% rapeseed meal (based on dry matter) and was provided in two different batches. Supplementation with FRS tended to lower FEC and reduce the incidence of *A. suum* infection. However, this effect varied between batches. FRS also influenced gut microbiota composition: pigs that were fed the second batch of FRS showed an increased relative abundance of several *Bacteroidetes* members (e.g., *Prevotella*), while unsupplemented pigs were enriched with various taxa from the *Firmicutes* phylum, such as *Clostridium* spp. Furthermore, pigs fed the second batch of FTR showed reduced daily weight gain compared to those fed the control diet. Overall, our results indicate that FRS modulates the gut microbiota toward a composition potentially associated with improved gut health and may have promise as a prebiotic or novel feed additive to help reduce helminth infections.

## 1 Background

Helminth parasites—primarily the pig roundworm *Ascaris suum* and the nodular worm *Oesophagostomum* spp.—are common in pig production systems worldwide, particularly in pigs raised outdoors (1). The eggs of *A. suum* can survive for years on pastures (2, 3), whereas the external stages of *Oesophagostomum* spp. are more sensitive to environmental conditions such as drought and frost (4). In northern temperate climates such as Scandinavia, larvae of *Oesophagostomum* spp. rarely survive outdoors during typical winters (5). As porcine helminths primarily cause subclinical disease, infections are often not perceived by producers as a threat to productivity (1). Nonetheless, negative correlations between helminth infections and pig growth or feed efficiency have been documented for both *A. suum* (6–9) and *Oesophagostomum* spp. (10, 11). Infections with the protozoa, *Eimeria* spp. are also common in weaned pigs. While usually non-pathogenic (12), the long-term effects of *Eimeria* infections on performance remain poorly understood (13).

Infections with *A. suum* in pigs have been associated with significant changes in the host's gut microbiota (GM) composition and induction of strong local immune responses (14). Thus, helminth infections are considered to influence animal welfare and health and lead to economic loss for producers, with frequent anthelmintic treatment being the obvious solution to limit helminth infections (1). However, due to the risk of anthelmintic resistance, as documented in *Oesophagostomum* spp. (15–18). There is a need for alternative approaches (14, 19). Resistance has also been documented for ascarid helminths in other hosts, e.g., turkeys, horses, and humans (20–22).

The search for alternative treatments has renewed interest in botanicals with anthelmintic properties (23). In ruminants, numerous such botanicals have been identified, including pasture species such as sainfoin and chicory (24, 25) as well as feed additives containing essential oils (26). In contrast, research on botanical alternatives for controlling pig parasites remains limited (27).

However, some evidence suggests that dietary additives such as garlic may hold promise as alternative anthelmintic agents (28). In addition, bioactive compounds—particularly omega-3 fatty acids such as alpha-linolenic acid—from extracts of brown seaweed (*Saccharina latissima*) have shown direct anti-parasitic effects *in vitro* against the larval stages of *A. suum* (29) and the canine hookworm *Uncinaria stenocephala* (30). However, Bonde et al. (31) were unable to demonstrate a consistent anti-parasitic effect in two short-term studies with pigs experimentally infected with *O. dentatum* and *A. suum* and fed a high level of dried whole *S. latissima* (5 or 8% of dry matter intake). Brown seaweeds (BS; *Phaeophyta*) are a recognized source of bioactive compounds, such as high concentrations of soluble polysaccharides (32–34). These complex polysaccharides are not digested and absorbed in the small intestines of the host and are instead fermented in the hindgut by the GM (33, 35). BS polysaccharides have shown anti-infection, anti-inflammatory, and immune-modulating properties in a variety of animal species (35, 36) and could potentially also have anti-parasitic properties, as shown for other prebiotic compounds in animals (37, 38). Furthermore, extracts of *Ascophylum nodosum* and other BS have shown promising results in the sustainable control of plant-parasitic nematodes, e.g., *Meloidogyne* spp., in a range of crops (39).

Brown seaweed species such as *S. latissima* and *A. nodosum* have been tested in combination with rapeseed (*Brassica napus*) in feed supplements for pigs (40), where rapeseed has been linked to improved gut health (41). However, as both seaweed and rapeseed contain anti-nutritional components (42, 43), a pre-fermentation process may improve the nutritional value, digestibility (44) and suitability of this product as a pig feed (45) and thereby increase growth performance (46). Pre-fermented rapeseed-seaweed supplements fed to piglets have thus been shown to increase GM diversity, reduce intestinal inflammation, and enhance gut mucosal development (40, 45, 47). Thus, based on these findings and *in vitro* findings, we hypothesize that long-term consumption of such supplements may help limit intestinal parasite infections; however, to date, no studies have examined the potential anti-parasitic effects of a pre-fermented rapeseed–seaweed mix fed to pigs under large-scale, on-farm conditions.

Here, we evaluated the effects of a 2% (w/w) supplementation with a pre-fermented rapeseed-seaweed product (FRS) in a standard pelleted diet on naturally acquired parasite infections (predominantly *A. suum*) and gut health in growing finishers, as measured by changes in GM composition and systemic inflammatory parameters.

## 2 Materials and methods

### 2.1 Experimental animals and design

In total, 200 weaners/early growers (Landrace/Yorkshire/Duroc crossbreeds) from a conventional indoor pig unit were introduced, 50 animals at a time, to a free-range farm at four time points during winter season (sub-studies 1–4): November 18th, 2020 (SUB1); January 6th 2021 (SUB2); February 3rd 2021 (SUB3); and February 17th 2021 (SUB4). At arrival, the pigs in each sub-study (*n* = 50) were randomly allocated to two groups of 25 animals after stratification for sex (females or castrated males; Table 1). One group received a standard control diet (group C), while the second group (group S) received a similar diet supplemented with 2% (w/w) FRS. Each group was housed in an individual paddock with a shed, feeding trough, and water system for the fattening period (~11–12 weeks) until slaughter weight was reached. The two feeding groups (control vs. FRS) within a sub-study were kept on neighboring paddocks, which were naturally contaminated with *Ascaris suum* and other parasites from earlier production. Paddocks were not re-used within the current study. All animals were weighed upon arrival, post-group allocation (week 0) and before slaughter (week 11–12). Animals were monitored daily by farm personnel. The study was conducted after approval by the Danish Animal Experimentation Inspectorate (license number 2015-15-0201-00760). Weather data were obtained from the Danish weather service (DMI) from the weather archive.

**Table 1:** Characteristics of sub-studies and all sub-studies combined (bold figures), in regard to dietary treatments, number of pigs included at the beginning and end of the study, as well as sex distribution (F = females), number of days in the trial, starting live weights and daily weight gain (DWG).

**Sub-study**	**Group**	**Diet**	***n* (start)**	**Sex (%F)**	***n* (end)**	**Trial days**	**Starting weight Mean ±SD (Kg)**	**DWG Mean ±SD (kg)**
SUB1	C1	Control	25	52%	24	84^*^	29.8 ± 1.8	1.09 ± 0.09
	S1	FRS (batch 1)	25	52%	24	84^*^	29.9 ± 1.8	1.06 ± 0.06
SUB2	C2	Control	25	56%	25	79	26.7 ± 2.0	1.06 ± 0.11
	S2	FRS (batch 2)	25	56%	24	79	27.7 ± 2.2	1.05 ± 0.08
SUB3	C3	Control	25	44%	23	78	28.1 ± 2.3	1.05 ± 0.09
	S3	FRS (batch 2)	25	40%	24	78	27.5 ± 1.8	1.02 ± 0.09
SUB4	C4	Control	25	44%	25	78	29.8 ± 2.1	1.12 ± 0.09^a^
	S4	FRS (batch 2)	25	44%	23	78	29.7 ± 1.4	1.05 ± 0.10^b^
SUB1–4	**C1–4**	Control	**100**	**49%**	**97**	–	**28.6** **±2.4**	**1.08** **±0.10**
	**S1–4**	FRS	**100**	**48%**	**95**	–	**28.7** **±2.1**	**1.05** **±0.09**

^*^Five pigs from both C1 and S1 were sent for slaughter after 79 days.

Different superscripts (^a, b^) indicate significant differences (P < 0.05) between control and FRS groups within the same sub-study.

### 2.2 Diets

All animals were fed *ad libitum* and had free access to water. C-groups were fed a basal commercial diet (Flex A, complete feed, DLG, Denmark mix) from three batches of similar composition and metabolizable energy [#644690 (SUB1+SUB2+SUB3), #665655, and #681221 (SUB2+SUB3+SUB4)], with a smooth transition between batches. S-groups were fed a similar commercial diet with 2% w/w inclusion of the FRS supplement, which is commercially available (EP1199; provided by Fermentationexperts A/S, Denmark). The inclusion level of the supplement was decided based on previous experience by the company providing it and cost-efficiency. Two different fermentation batches of the FRS supplement diet were produced. Batch 1 was used for SUB1, and Batch 2 was used for SUB2–4. The two FRS supplement batches contained 6% *Saccharina latissima*, 6% *Ascophylum nodosum*, and 88% rapeseed [based on dry matter (DM)], which were processed and fermented as described elsewhere (40, 45, 47). Briefly, the fermentation was a solid-state fermentation using an inoculum based on three lactic acid fermentation bacteria: *Pediococcus acidilactici* (DSM 16243), *Pediococcus pentosaceus* (DSM 12834), and *Lactobacillus plantarum* (DSM 12837). The fermentation was a two-step process at 38°C and took a total of 11 days, followed by drying in a spin flash dryer. Both control and FRS diets were pelleted and adjusted to similar levels of protein and energy content (Table 2).

**Table 2:** Main ingredients and composition analysis of control diet and experimental diet with 2% w/w fermented rapeseed-seaweed (FRS) supplement.

**Ingredients (%)**	**Control**	**FRS batch 1**	**FRS batch 2**
Barley	35.79	35.00	35.00
Wheat	25.00	26.31	26.31
Soybean meal	10.47	10.71	10.71
Wheat bran	6.10	–	–
Rye	5.30	10.00	10.00
Oats	4.00	2.00	2.00
Sunflower meal	7.23	8.00	8.00
FRS^*^	–	2.00	2.00
Dried sugar beet	2.00	2.00	2.00
Feed chalk	–	1.17	1.17
Vegetable oil and fat (palm)	0.90	1.01	1.01
Calcium format	0.50	–	–
Calcium carbonate	0.80	–	–
Mono calcium phosphate	0.20	0.31	0.31
Sodium chloride	0.54	0.48	0.48
L-lysine sulfate	0.60	0.59	0.59
Vitamin mix	0.20	0.23	0.23
**Analyzed composition**			
Crude fat (%)	3.2		3.2
Crude protein (%)	16.1		16.3
Water (%)	12.2		12.9
Crude ash (%)	4.8		4.9
Crude fat (%)	3.2		3.2
Metabolisable energy (MJ/kg)	13.22		13.15

^*^FRS, fermented rapeseed-seaweed.

### 2.3 Fecal egg counts

Fecal samples were initially collected in weeks 0, 5, 7, 9, and 11 or 12 for SUB1 and SUB2 for individual fecal egg counts (FEC) to characterize parasite transmission patterns based on eggs per gram of feces (EPG). Thereafter, SUB3 and SUB4 were only sampled in weeks 0, 9, and 11. The samples were analyzed using a modified concentration McMaster method (2, 48) with a lower threshold of 20 EPG. Feces were sampled directly from the rectum.

### 2.4 Antibodies and cytokines

Blood samples were collected from all animals via jugular venipuncture using SST™ II Advance BD Vacutainer tubes at week 11 (SUB2) or week 12 (SUB1) for antibody detection and cytokine analysis. Samples were centrifuged at RCF 1,000 for 15 min, and serum was stored at −80°C until use. Serum samples were analyzed for specific IgG antibodies against *A. suum* adult hemoglobin [As-Hb; cut-off Optical Density ratio (ODr) = 0.5] and antigens from third-stage larvae isolated from the lungs (cut-off ODr = 0.25) using the ELISA as previously described (49). Further, concentrations of the inflammatory cytokines IL-6 and TNF-α in serum were assessed using commercial antibody pairs (R and D Systems, UK) according to the manufacturer's instructions.

### 2.5 Gut microbiota analysis: DNA extraction, 16S rRNA gene amplicon sequencing, and data processing

The Bead-Beat Micro AX Gravity Kit (cat# 106-100-M1; A&A Biotechnology, Gdynia, Poland) was used to extract DNA from fecal samples from the experiments SUB1 (week 0 and 12) and SUB2–4 (week 0 and 11) based on the manufacturer's guidelines. DNA purity and concentration were determined using a Nanodrop 1000 Spectrophotometer (Thermo Fisher Scientific, USA) and Varioskan Flash (Thermo Fisher Scientific, USA).

A two-step PCR was carried out to amplify the near full-length 16S rRNA gene with multiple forward and reverse primers (Supplementary Table 1). Reaction conditions for the first PCR were as follows: 95°C for 5 min, two cycles of 95°C for 20 s, 48°C for 30 s, 65°C for 10 s, 72°C for 45 s, and a final extension at 72°C for 4 min. First PCR products were next barcoded by a second PCR reaction with the following conditions: 95°C for 2 min, followed by 33 cycles of 95°C for 20 s, 55°C for 20 s, 72°C for 40 s, and a final extension at 72°C for 4 min. After each PCR reaction, PCR products were cleaned using SpeedBeads™ magnetic carboxylate (obtained from Sigma Aldrich). A 1.5% agarose gel electrophoresis was performed to check the size of barcoded PCR products.

The Nanopore sequencing library was prepared following the ligation sequencing kit SQK-LSK109 and SQK-LSK110 protocols and sequenced on a GridIONX5 platform (Oxford Nanopore Technologies, Oxford, UK).

Sequence data collection was conducted using the Nanopore sequencing software GridION version 21.02.5 (https://nanoporetech.com). ONT's Guppy version 4.5.2 (https://nanoporetech.com) was used for base calling and demultiplexing. Demultiplexed sequences were filtered and trimmed (min = 1,300 bp, max = 1,600 bp, *q* score ≥10) using Nanofilt version 2.7.1 (50). Taxonomy assignment was conducted by the parallel_assign_taxonomy_uclust.py script of Quantitative Insights into Microbial Ecology (Qiime) 1 version 1.8.0 (51). The Greengenes database version 13.8 (52) was used as a reference database.

### 2.6 Statistical analysis

Data from eight animals were removed during the study (see Section 3.1 for listed reasons), and data were excluded from the analysis. Due to the change in FR's batches, all SUB1 data were analyzed separately, while SUB2–4 data were analyzed together. Infection levels were based on raw FEC, expressed as accumulated FEC based on the sum of egg excretion from each animal on sample days, whereas *A. suum* prevalence and incidence were determined based on FEC >200 EPG to estimate likely patent infections and exclude potentially false-positive pigs (53). Incidence refers to the number of initially uninfected pigs becoming infected during the study, i.e., the proportion of a group having a positive FEC (>200 EPG) at least once after initial sampling. All data were checked for normality using the Shapiro-Wilks test using GraphPad Prism (7.00). Data that could be normalized through log transformation were analyzed using an unpaired *t*-test, with Welch's correction applied where needed. Data that could not be normalized were analyzed using a Mann–Whitney test. A chi-squared or Fisher's exact test was performed using R Studio (R version 3.5.2) to estimate differences in infection incidence between groups for all three different parasites. Data were also analyzed using a linear model (LM, Type III) using R to test factors affecting DWG and *A. suum* FEC. The model included the infection status of *A. suum*, feed, sex, sub-study, and body weight at arrival (BW0) and was tested for interactions. The infection status of *Eimeria* spp. and *Oesophagostomum* spp. was not included due to sporadic, low-level infections. Similarly, effects on *A. suum* cumulative FEC were analyzed as a linear model for factors: sub-study, diet, sex, and DWG for weeks 9 and 11/12. The linear model for FEC was considered both with and without log transformation. The Spearman rank correlation test examined correlations between log-transformed FEC and antibody responses.

16S rRNA gene amplicon sequencing data were analyzed separately for SUB1 (*n* = 90) and SUB2–4 (*n* = 282). Data were rarefied to 11,000 reads per sample using QIIME 2 (54). After rarefaction, four samples from SUB1, three from SUB2, six from SUB3, and one from SUB4 were excluded from the analysis since they had fewer than 11,000 reads. RStudio version 1.3.1073 (55) using R version 4.0.2 (55) and R packages phyloseq (56), tidyverse (57), ggpubr (58), reshape2 (59), and ggprism (60) were used for data analysis. Observed features and Shannon index measures were calculated for alpha diversity evaluation. Beta diversity analysis was performed by generating principal coordinate analysis (PCoA) plots based on Bray-Curtis dissimilarity and Jaccard distances. Bacteria differentially abundant between groups were identified using the DESeq2 package (61), and a heatmap of taxa found to be significantly differently abundant between groups by DESeq2 (adjusted *P*-value < 0.05) was drawn by using the pheatmap (62) and RColorBrewer (63) packages.

For GM analysis, adjusted *P*-values for alpha diversity measures were obtained by pairwise Wilcoxon rank-sum tests with the Benjamini–Hochberg correction from the R stats package (55). Adjusted *P*-values for beta diversity were calculated by pairwise comparisons using permutation MANOVAs on a distance matrix with *P*-value correction using the Holm method from the R package RVAideMemoire (64).

## 3 Results

### 3.1 Observations and performance

The weather conditions varied during the study, as the average daily temperatures during SUB1, SUB2, SUB3, and SUB4 were 4.63, 2.27, 3.73, and 5.45°C, respectively (data not shown). February was particularly cold, thus affecting SUB2 and SUB3, with the lowest temperature during the study measured at −11.9°C, and the mean temperature for the month was 1.1°C. In March, the second lowest temperature was recorded at −6.2°C, with a mean for the month at 4.6°C, affecting the outcome of SUB2–4. Both diets were well accepted by the pigs. Eight pigs were removed from the study: five individuals died from unknown reasons (C1, S2, C3, S3, and S4), two pigs were withdrawn due to a rectal prolapse/stricture (S4 and C3), and one pig was excluded after it managed to jump the fence into another paddock (S1 → F0E0C1).

**Figure 1:**
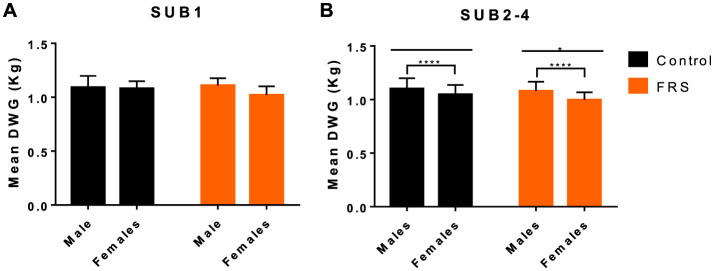
Mean (+SD) daily weight gain (DWG) in relation to diet [control and fermented rapeseed-seaweed (FRS)] and sex for **(A)** sub-study 1 (SUB1; FRS batch 1), and **(B)** combined for sub-study 2–4 (SUB2–4; FRS batch 2; ****P < 0.001, *P < 0.05, by linear model).

We found no difference in initial body weight between the feeding groups in each sub-study (Table 1) and between male (castrated) and female pigs (28.18 vs. 28.35 kg, *P* = 0.77). During the study, FRS-fed pigs tended to gain less weight than their respective control groups (Table 1), though only significantly in SUB4 (*P* = 0.02). Applying a linear model to SUB2–4 confirmed that mean daily weight gain (DWG) was significantly affected by diet (*P* < 0.01), sub-study, and sex, with males growing 0.07 kg/day more than females (*P* < 0.0001; Figure 1). Moreover, for SUB2–4, the model showed that pigs with patent *A. suum* infection tended to have a lower DWG than uninfected pigs (−0.03 kg/day; *P* = 0.11). There were no interactions between the parameters. For SUB1 sex, it did not significantly affect DWG, but male pigs tended to grow 0.05 kg/day more than female ones (*P* = 0.07). Within all four sub-studies, the largest difference between males and females was in the FRS groups (Figure 1). In summary, DWG was 0.04 kg/day lower for FRS-fed animals, but the difference was only significant in SUB4.

### 3.2 FRS supplementation did not significantly reduce parasite infections

**Figure 2:**
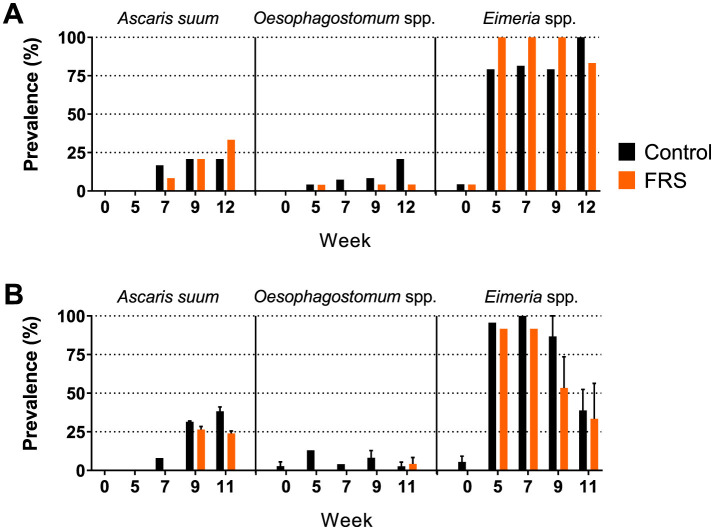
Prevalence of fecal egg/oocyst excretion of parasites in **(A)** sub-study 1 (SUB1; FRS batch 1) and **(B)** combined for sub-study 2–4 (SUB2–4; FRS batch 2). Data for the latter is presented as mean ± SEM (weeks 5 and 7 only include sub-study 2).

**Figure 3:**
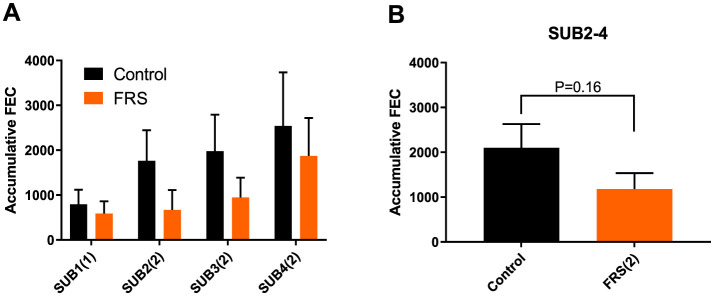
Mean (± SEM) accumulated fecal egg count (FEC) of *Ascaris suum* in **(A)** each sub-study (SUB1–4) fed either FRS batch 1 or 2, indicated by (number) in brackets and **(B)** in combined sub-studies fed FRS batch 2, i.e., SUB2–4. Data are represented as mean ± SEM with *P*-values (ANOVA).

**Figure 4:**
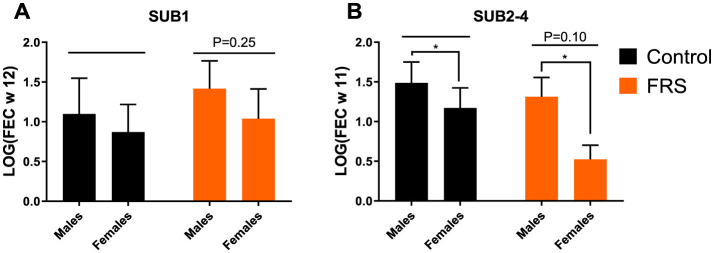
Fecal egg count (FEC), based on logarithmic transformation [log(FEC)] for week 12 for *Ascaris suum* of **(A)** sub-study 1 (SUB1), and **(B)** combined for sub-study 2-4 (SUB2–4) week 11. Data are represented as mean ± SEM with *P*-values (ANOVA; *P-value < 0.05, by linear model).

Eggs of *A. suum* and *Oesophagostomum* spp., as well as *Eimeria* oocysts, were detected in the fecal samples. Due to sporadic detection, data on *Cystoisospora suis* were excluded from further analysis. Based on the sampling schedule for SUB1 and SUB2, patent *A. suum* egg excretion (i.e., >200 EPG) was first detected in week 7 (Figure 2), indicating that the infections were acquired on pastures and not in the herd of origin. Patent *A. suum* infections occurred in all subgroups (Table 3). Specifically, in SUB1, the prevalence was initially lower for the FRS group in week 7 but was higher in week 12 than in the control group (Figure 2). In contrast, the FRS groups in SUB2–4 consistently had a lower prevalence than the control groups at all time points. This was also reflected in lower incidence levels in FRS groups compared to controls in SUB2–4 (Table 3), although the difference was not statistically significant (*p* = 0.19). With respect to the accumulated *A. suum* FEC, calculated as the sum of egg excretion from each individual animal across sample days, the FRS groups in all four sub-studies excreted fewer *A. suum* eggs overall compared to the control groups; however, the difference was not statistically significant. The outcome was similar (*P* = 0.16) when combining SUB2–4, even though the total accumulated egg excretion by FR's pigs was 45.3% lower than that of control pigs (Figure 3). In SUB1, the total accumulated egg excretion was 29.1% lower in FRS-fed animals. The linear model identified that SUB2–4 FECs at weeks 9 and 11 were significantly influenced by sex (Figure 4, Supplementary Table 2), with diet trending toward significance in weeks 9 and 11 (*P* = 0.08 and *P* = 0.10, respectively). However, FEC in SUB1 was not affected by either diet or sex (*P* > 0.15) despite lower excretion in females compared to males (Figure 4).

**Table 3:** Incidence, i.e., new infections picked up during the whole study, and fecal egg excretion accumulated over sample days (Acc. FEC) of parasites of individual groups and for sub-study 2–4 combined (figures in bold).

**Sub-study**	**Group**	***Ascaris suum***		***Oesophagostomum*** **spp**.		***Eimeria* spp**.
		**Incidence**	**Acc. FEC (epg)**	**Incidence**	**Acc. FEC (epg)**	**Incidence**
SUB1	C1	20.8%	19,860	29.2%	220^a^	100.0%
	S1	33.3%	14,090	8.3%	30^b^	100.0%
SUB2	C2	40.0%	44,150	24.0%	8,150	100.0%
	S2	25.0%	16,040	12.5%	50	100.0%
SUB3	C3	34.8%	45,485	8.7%	20	100.0%
	S3	25.0%	22,780	0.0%	0	83.3%
SUB4	C4	36.0%	63,580	0.0%	0	60.0%^a^
	S4	30.4%	44,931	0.0%	0	21.7%^c^
**SUB2–4**	**C2–4**	**37.0%**	**153,215**	**11.0%**	**8,170**	**86.3%** ^**a**^
	**S2–4**	**26.8%**	**83,751**	**4.2%**	**50**	**69.0%** ^**c**^

Different superscripts (^a, b, c^) indicate significant differences (a–b: P = 0.05, a–c: P = 0.01) between control and FRS groups within the same sub-study.

*Oesophagostomum* spp. egg excretion was not detected in SUB3 and SUB4, except for two animals with low egg counts (10 EPG) in group C3, week 9, and the incidence rates were generally low (Table 3). No significant difference in incidence rates between diet groups was thus found within SUB1 (*P* = 0.14) or SUB2–4 (*P* = 0.13). The total accumulated FEC was significantly lower in the FRS group than in the control group in SUB1 (*P* = 0.05) and showed a trend of being lower in the FRS group than in the control group in SUB2 (non-significant). However, the higher accumulated FEC count in the C2 group compared to the S2 group was likely due to an outlier—one pig that exhibited a high FEC of 6,600 EPG at week 0.

*Eimeria* spp. were detected in pigs from all groups after arrival (Table 3), with incidences much higher in SUB1–3 than SUB4 for both diet groups. The incidence was significantly lower for FRS pigs compared to controls for SUB2–4 (*P* = 0.01), which likely reflects a large difference in incidence within SUB4 (Table 3). Only a few oocysts were present in positive pigs in SUB4.

In general, we found that FRS inclusion significantly reduced or showed tendencies of reduced incidence of infection for all three parasites and tendencies of reduced FEC and prevalence each week for both *A. suum* and *Oesophagostomum* spp. However, we again saw a batch difference in that FRS batch 1 in SUB1 increased *A. suum* incidence and prevalence.

### 3.3 High anti-*A. suum* antibody levels in all groups (SUB1 and SUB2)

All animals were highly positive for specific antibodies against both adult and larval *A. suum* in these sub-studies, even pigs not excreting *A. suum* eggs. Thus, there was no correlation between ODr values and FEC for SUB1 or SUB2. There were also no significant differences in As-Hb OD values (Supplementary Figure 1). However, S1 had significantly lower ODr values for antibodies against larval antigens compared to C1. Furthermore, no significant differences were found in the levels of IL-6 and TNF-α (data not shown). Serum samples from SUB3 and SUB4 were not analyzed.

### 3.4 FRS but not parasite infection levels modulate gut microbiota diversity and composition

**Figure 5:**
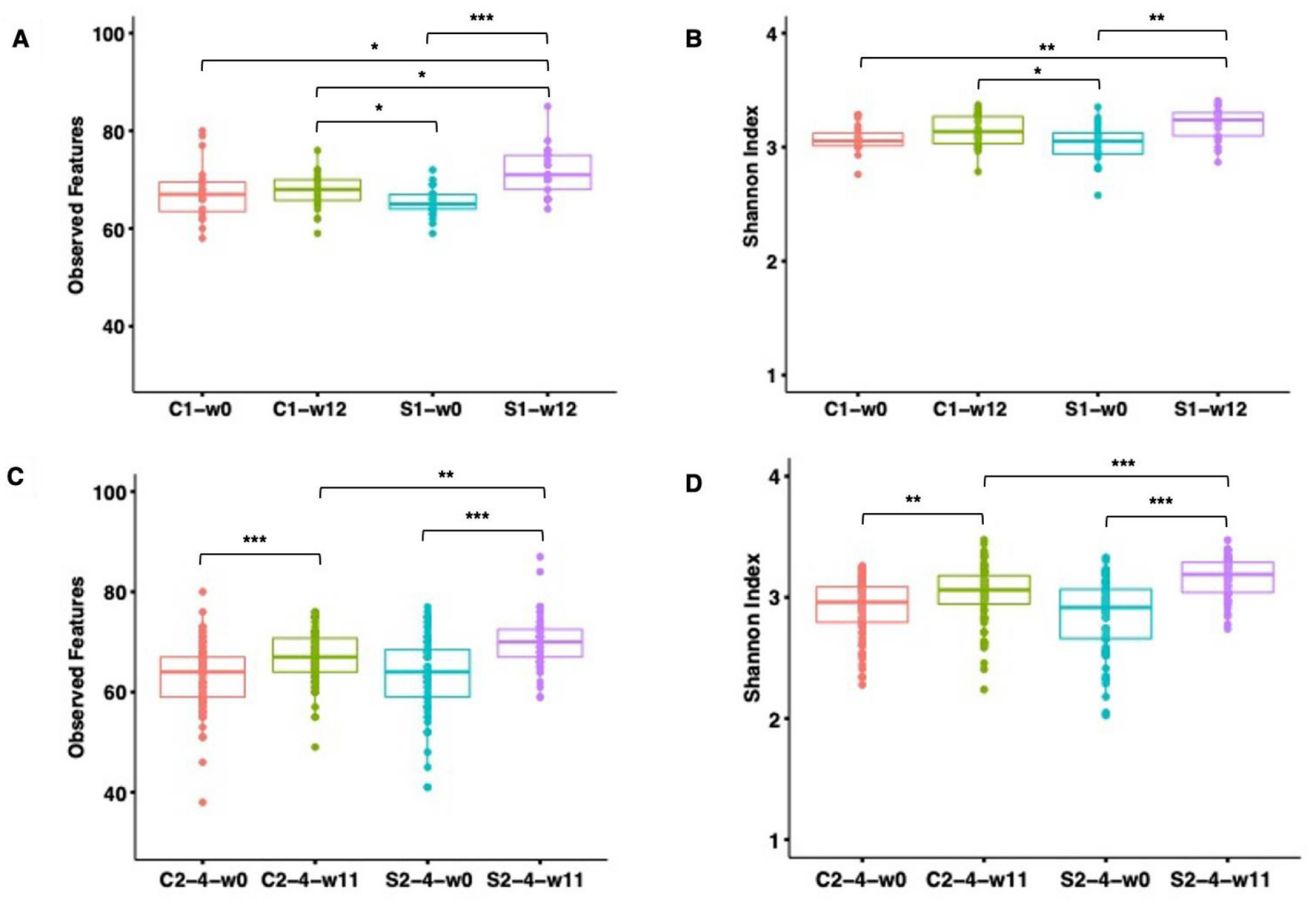
Observed species and Shannon diversity index of the gut microbiome of pigs fed with FRS-supplemented diet (S1 and S2–4) and control diet (C1 and C2–4). Boxplots illustrate alpha diversity for SUB1 based on observed features (species) **(A)** and Shannon index **(B)** and for SUB2–4 based on observed features (species) **(C)** and Shannon index **(D)**. Each box represents the interquartile range between the 25th and 75th quartiles, and the horizontal line inside the boxes shows the median. The significance of the difference in alpha diversity was assessed by pairwise Wilcoxon rank-sum test with Benjamin–Hochberg correction. The level of significance was marked with stars on the plot (***P ≤ 0.001, **P ≤ 0.01, *P ≤ 0.05). No star was flagged on the plots if P > 0.05.

The microbial community richness and diversity were determined in SUB1 and SUB2–4 based on two alpha diversity indices, i.e., observed features and the Shannon index (Figure 5). Before starting the diet intervention, baseline alpha diversity indices did not significantly differ between C- and S-groups at week 0 for SUB1 and SUB2–4 (*P* > 0.05). In SUB1, feeding the control diet did not result in any significant changes in alpha diversity indices over time (*P* > 0.05), while we observed a significant increase in observed features (*P* = 0.0002), and the Shannon index (*P* = 0.004) over time after FRS diet feeding. In SUB2–4, we observed increases in both alpha diversity indices over time for pigs fed both control feed and the FRS diet (*P* < 0.01). In addition, we examined the FRS intervention effect by comparing C and S groups at week 12 or 11. We found that FRS intervention increased the number of observed features in SUB1 (*P* = 0.02), both observed features (*P* = 0.002) and the Shannon index in SUB2–4 (*P* = 0.0008).

**Figure 6:**
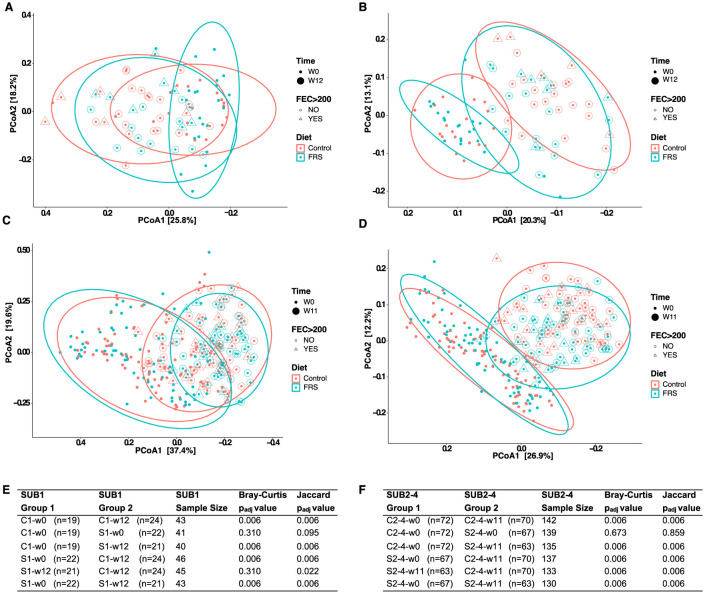
Development in gut microbiota composition of pigs fed with FRS supplemented diet and control diet. Principal coordinates analysis (PCoA) based on **(A, C)** Bray-Curtis dissimilarity (left) and **(B, D)** Jaccard distances (right) were performed for SUB1 (upper panel) and SUB2 (lower panel). Each data point on PCoA plots indicates samples that are depicted based on diet, FEC >200, and time. Ellipses denote a 95% confidence interval, and the percentage in brackets is the percentage of variation explained by each PCoA axis. Statistical pairwise group comparisons for beta diversity using permutation MANOVAs on a distance matrix with *P*-value correction using the Holm method for **(E)** SUB1 and **(F)** SUB2–4. *P* < 0.05 was considered as significant.

Subsequently, Bray–Curtis dissimilarity metrics (Figures 6A, C) and Jaccard (Figures 6B, D) distances were determined to investigate the influence of time, feed, and parasites on the overall GM composition. Bray–Curtis dissimilarity metrics showed, as expected, no clustering according to diet at baseline (*P* > 0.05). On both diets, the GM significantly changed over time (*P* = 0.006 for both Bray-Curtis and Jaccard). However, the FRS supplementation had different effects in SUB1 and SUB2–4. We observed a significant FRS intervention effect based on Jaccard in SUB1 (*P* = 0.022) (Figure 6E), whereas this effect was also significant in SUB2–4, both based on Bray–Curtis and Jaccard (*P* = 0.006) (Figure 6F).

We next analyzed beta diversity in C and S groups at week 12 or 11, according to their infection status. We found that infection affected only the GM composition within the different feeding groups based only on Jaccard distances in SUB1 (C1-w12-infection vs. C1-w12-no-infection; *P* = 0.05) and SUB2–4 (C2–4-w11-infection vs. C2–4-w11-no-infection and S2–4-w11-infection vs. S2–4-w11-no-infection; *P* = 0.02).

**Figure 7:**
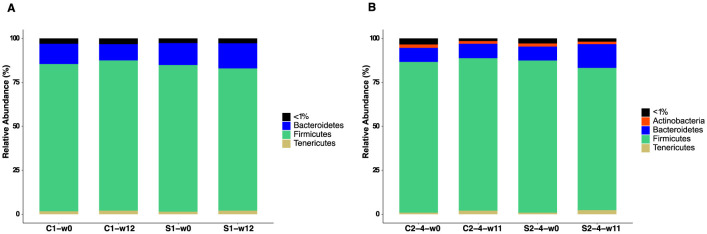
Phylum-level relative abundance of gut microbiota in pigs fed with FRS supplemented diet (S1 and S2–4) and control diet (C1 and C2–4). Stacked bar plots show the phyla with a median relative abundance above 1% for **(A)** SUB1 at weeks 0 and 12 and **(B)** SUB2–4 at weeks 0 and 11. “ <1%” represents the phyla in each group with a median relative abundance below 1%.

To assess bacterial GM composition, we initially analyzed the phyla with a median relative abundance >1% in each group of SUB1 and SUB2–4 (Figure 7). We found *Firmicutes* and *Bacteroidetes* to be the most abundant phyla in all groups, with *Tenericutes* also detected in most pigs. Moreover, the phylum *Actinobacteria* was present in SUB2–4 but not in SUB1.

We finally assessed differentially abundant bacteria by performing DESeq2 analysis at the species level. Prior to diet intervention, only one bacterium significantly differed in the baseline GM of pigs in SUB1 (Supplementary Figure 2), and there was no significant difference in baseline in SUB2–4. In line with the observations above, DESeq2 analysis also showed that the effect of including FRS in the feed differed between SUB1 and SUB2–4. Only *Bifidobacterium thermacidophilum* was more abundant in the C-group than the S-group at week 12 in SUB1 (Supplementary Figure 3A). But, in SUB2–4, 18 bacterial species significantly differed in abundance by week 11 (Supplementary Figure 3B). In SUB2–4, FRS led to increased relative abundance of a range of *Bacteroidetes* members, e.g., *Prevotella* and S24-7, while the unsupplemented pigs were enriched in various *Firmicutes*, such as *Clostridium* and *Turicibacter* spp.

In general, we found that the FRS supplementation in SUB2–4 (batch 2) induced major alterations to the GM compared to controls, contrasting with the more moderate changes in SUB1 with the FRS from batch 1.

## 4 Discussion

This large on-farm study, which investigated the effects of 2% FRS dietary inclusion on naturally acquired parasite infections and GM composition, showed a clear tendency of reduced incidence of infection of parasites in SUB2–4 using the FRS batch 2. The pigs were primarily infected with *A. suum*, and all sampled pigs (both control and FRS-fed pigs) were highly positive for antibodies against *A. suum*, indicating a high level of exposure on all pastures in SUB1 and SUB2. Furthermore, we found increased diversity in the microbiota of FRS-fed pigs compared to control-fed pigs; however, we also observed a decrease in daily weight gain (DWG). As this was a field study and pasture infectivity levels were not quantified, we cannot exclude the possibility that varying infection pressures at the start may have influenced the results. However, we consider this unlikely, as paddocks were randomly allocated to the different dietary treatments, and the study was replicated across multiple substudies.

The decreased incidence and reduced FEC indicate that FRS may contain anthelmintic phytochemicals. We previously found that *S. latissima* extracts killed >90% of third-stage *A. suum* larvae *in vitro* (29), supporting the idea that bioactive compounds in brown seaweed may contribute to the observed antiparasitic effects in this study. Thus, we observed tendencies toward reduced accumulated FEC and incidences of *A. suum* (SUB2–4) and *Oesophagostomum* spp. (SUB1 and SUB2), and *Eimeria* spp. (SUB3 and SUB4), suggesting that inclusion of FRS in the diet may exert an anti-parasitic effect, potentially by affecting parasite establishment and/or fecundity. In addition to direct anti-parasitic activity, FRS may also improve gut barrier integrity and mucosal immune responses, thereby supporting host defense against infection (40, 65). Further studies will be necessary to elucidate these potential mechanisms of action. Moreover, due to the generally low prevalence and infection levels observed, it remains inconclusive whether FRS had a definitive impact on O*esophagostomum* spp. infections.

The general declining incidence levels observed for *Oesophagostomum* spp. and *Eimeria* spp. from SUB1 to SUB4 during winter are most likely due to weather conditions since SUB3 and SUB4 were introduced in February 2021 (3rd and 17th), which was the coldest month recorded during this study. Eggs and free-living larvae of *Oesophagostomum* spp. are considered very susceptible to extreme weather conditions (4, 5). A reduction in sporulation has been reported for the porcine *E. debliecki* and *E. scabra* exposed to freezing, thawing, and continuous freezing (66). Moreover, freezing has been found to inactivate chicken *Eimeria* spp. (67) and reduce bovine *Eimeria* spp. oocysts when kept at −18°C (68). In contrast, the transmission of *A. suum* appeared unaffected by weather conditions during this study, as judged by accumulated FEC over the sub-studies. *A. suum* eggs are very resistant to environmental conditions (2), especially when unembryonated, and can survive freezing temperatures down to −27°C for 10 days when containing embryos, although freezing usually diminishes infectivity (69). Due to low temperatures during the study, it is unlikely that any embryonation of excreted eggs took place in the paddocks.

All tested pigs were positive for specific *A. suum* serum antibodies at the end of the study, even though they did not all have patent infections. This indicates that all pigs were exposed to the parasite on the pastures, but that acquired immunity resulted in the expulsion of the parasites before the infections reached patency (70). Additionally, some low infection levels may have remained undetected. Studies have shown that worm burdens are dose-independent and highly variable, as 10% of pigs may harbor 80% of the burden (71).

The seroprevalence of *A. suum* infections is normally found to be higher than coproprevalence (49, 72), as in our study with 100% seroprevalence. However, our results question whether these serology tests can overestimate infection prevalence within a farm since the antibody response only indicates exposure level and not individual patent infections (72). A positive correlation between FEC and anti-AsHb-IgG antibodies has been established (49), as well as in naturally infected weaners and fatteners (72). However, this was not present in our study, and other studies found no correlation between ELISA OD levels and worm burdens in naturally infected pigs (73).

Prebiotics have also been recognized to have a significant impact on helminth infections (38), as observed for *Oesophagostomum* spp. repeatedly (37) and *Trichuris suis* occasionally (74). The effects of FEC and transmission could be due to interactions with the microbiota or a direct effect by fermentation products (75) since both BS and rapeseed meal have been shown to contain compounds with prebiotic properties (76–78).

The fecal microbiota analysis showed overall large changes during the 11–12 weeks of the two sub-studies, which is in agreement with previous observations (79). The inclusion of FRS in the feed in our study influenced GM composition in all sub-studies, but it had an overall stronger effect in SUB2–4. This effect of FRS to modulate the pig GM is also in agreement with previous studies (40, 45), including our previous study with pigs co-infected with *A. suum* and *Oesophagostomum* (31). FRS contains various compounds influencing microbiota, such as polysaccharides, glycosinolates, and other bioactives. The difference in the effects on the microbiota of FRS diet inclusion in SUB1 and SUB2–4 indicates the batches were not completely similar. Fermentation is a complex biological process, and batch-to-batch variation occurs randomly or if variables such as temperature, pressure, pH, dissolved oxygen, and feed flow are not thoroughly controlled (80).

While the effect of FRS inclusion on specific GM members (as determined by DESeq2 analysis) was subtle in SUB1, the effect was considerably stronger in SUB2–4, where FRS was associated with increased relative abundance of mainly *Bacteroidetes* members such as *Prevotella* spp. Members of this genus are potent degraders of various undigestible carbohydrates and may thereby stimulate the ability to extract energy from feed components (79). Notably, *Prevotella* abundance has consistently been shown to increase in response to supplementation with seaweed-derived polysaccharides in pigs (81), calves (82), and bulls (83). *Prevotella* spp. have also been associated with increased fat accumulation in Duroc pigs; however, the implications of this relationship for gut health in pigs remain unclear (84). In contrast to this study, Bonde et al. (31) showed no overall change in *Bacteroidetes* but a decrease in specifically *Prevotella copri* in both infected and uninfected animals fed a substantially higher level of fermented *S. latissima* (8% dry weight).

In one sub-study (SUB4), the FRS diet had a significant negative impact on weight gain. The FRS feed had a slightly lower metabolizable energy (−0.5%) than the control feed, but probably not enough to explain the differences in weight gain (−3%). A negative effect of FRS on DWG may be related to the effects on microbiota, lower palatability, or anti-nutritional factors in both the rapeseed (41) and BS (40). Rapeseed has a high content of condensed tannins (6% in rapeseed hulls) (85). Fermentation of FR's supplement has been applied to mitigate anti-nutritional effects and has been shown to generally increase the weight performance in growing finishers (44, 46, 47). However, as tannins may form complexes with macromolecules such as proteins (86), they may reduce the overall digestibility of the feed (87). In our case, fermentation may not have been sufficient to eliminate all anti-nutritional factors. Furthermore, tannin–protein complexes have been coupled to a bitter taste (88), which could have reduced the feed intake and, thus, the weight gain of the pigs. It also remains speculative whether rapeseed or seaweed is responsible for anti-nutritional effects. The inclusion of seaweeds (*S. latissima* and *A. nodosum*) in the supplement to weaners indicated that the seaweed inclusion may have imposed a negative effect on weight gain compared to weaners fed fermented rapeseed alone (45). This may be partly caused by the high content of fermentable/indigestible polysaccharides of BS (34, 89) since growing finishers have a fully developed hindgut around the end-time of the fattening period (79), but in weaner piglets their hindgut is not yet well developed for fermentation (90).

That castrated male pigs grow better than female pigs in our study has previously been seen for growing finishers, with males also having a higher daily feed intake (91, 92). However, in other studies, the growth between male and female finishers was similar, though males did have a significantly better feed conversion ratio than females (93, 94). The current higher DWG in males may explain part of the observed higher egg excretion in males, as larger animals generally are considered to have a higher ingestion of parasite eggs and a larger intestinal surface area to host them (95). However, hormonal differences cannot be ruled out (96).

## 5 Conclusion

Trends in *A. suum* infection dynamics indicated that the inclusion of a 2% FRS supplement in the basal diet had a moderate effect, reducing egg excretion by 45% and lowering the number of new infection cases. This indicates that the FRS product may, depending on the batch, contain anthelmintic phytochemicals capable of reducing nematode infection levels. The 2% FRS supplement also modulated GM composition, notably increasing the relative abundance of *Prevotella* spp., but was associated with a slight reduction in weight gain. Thus, while the observed modulation of GM composition supports the potential use of FRS as a prebiotic dietary supplement, a deeper understanding of the mechanisms underlying its effects on gut function is needed to refine this strategy. Such refinement is essential to ensure health benefits while optimizing productivity. Furthermore, standardization of the production process to achieve consistent bioactivity across FRS batches will likely be crucial to realizing its full potential in future applications.

## Data Availability

The datasets presented in this study can be found in online repositories. The names of the repository/repositories and accession number(s) can be found at: https://www.ncbi.nlm.nih.gov/, PRJNA854963.

## Supplementary material

Supplementary Table 1 UMI containing multiple forward and reverse primers used in this study.

Supplementary Table 2 P-values for the final model of fecal egg count (FEC), based on logarithmic transformation for weeks 9 and 12 or 11. Includes Sub-study 1 (SUB1) and combined Sub-studies 2–4 (SUB2–4). P-values are from the final model, whereas “Excl.” refers to factors excluded from the final model (>0.15).

Supplementary Figure 1 Mean (+ SD) optical density ratio (ODr) as measurement of serum IgG antibodies against adult (As-Hb) and larvae (L3 lung) Ascaris suum in pigs from for **(A)** sub-study 1, group C1 and S1; **(B)** Response when divided into FEC positive or FEC negative based on FEC >200 for sub-study 1; and **(C)** sub-study 2, group C2 and S2; **(D)** Response when divided into FEC positive or FEC negative based on EPG >200 for sub-study 2 (^****^P <0.0001). The stippled line represents the cut-off value for As-Hb (0.50) and L3 lung test (0.25).

Supplementary Figure 2 Heatmap of bacteria found to be significantly differently abundant by DESeq2 analysis for SUB1 at week 0. The heatmap shows the comparison of control-fed (C) and FRS-fed (S) groups. Significantly different bacteria (Adjusted *P*-value < 0.05) at the species level are demonstrated in the heatmap.

Supplementary Figure 3 Heatmap of bacteria found to be significantly differently abundant by DESeq2 analysis for **(A)** SUB1 at week 12 and **(B)** SUB2–4 at week 11. Heatmap shows the comparison of control-fed (C) and FRS-fed (S) groups for **(A)** SUB1 and **(B)** SUB2–4. Significantly different bacteria (Adjusted *P*-value < 0.05) at the species level are demonstrated in heatmaps.

## References

[1] Roepstorff A Mejer H Nejsum P Thamsborg SM. Helminth parasites in pigs: new challenges in pig production and current research highlights. Vet Parasitol. (2011) 180:72–81. 10.1016/j.vetpar.2011.05.029 21684689

[2] Roepstorff A Nansen P. Epidemiology, diagnosis and control of helminth parasites of swine. FAO Anim Health Man. (1998) 3:171.

[3] Mejer H Roepstorff A. Long-term survival of *Ascaris suum* and *Trichuris suis* eggs in relation to pasture management. In: Proc 23rd Int Conf World Assoc Adv Vet Parasitol. Buenos Aires (2011). p. 113.

[4] Rose JH Small AJ. Observations on the development and survival of the free-living stages of *Hyostrongylus rubidus* both in their natural environments out-of-doors and under controlled conditions in the laboratory. Parasitology. (1982) 85 (Pt 1):33–43. 10.1017/S0031182000054123 7122126

[5] Roepstorff A Murrell KD. Transmission dynamics of helminth parasites of pigs on continuous pasture: *Oesophagostomum dentatum* and *Hyostrongylus rubidus*. Int J Parasitol. (1997) 27:553–62. 10.1016/S0020-7519(97)00023-4 9193949

[6] Hale OM Stewart TB Marti OG. Influence of an experimental Infection of *Ascaris suum* on performance of pigs. J Anim Sci. (1985) 60:220–5. 10.2527/jas1985.601220x 3972742

[7] Bernardo TM Dohoo IR Donald A. Effect of ascariasis and respiratory diseases on growth rates in swine. Can J Vet Res. (1990) 54:278–84. 2357666 PMC1255650

[8] Thamsborg SM Nejsum P Mejer H. Chapter 14 - Impact of *Ascaris suum* in livestock. In:Holland C, editors. Ascaris: The Neglected Parasite. Amsterdam: Elsevier (2013). p. 363–81. 10.1016/B978-0-12-396978-1.00014-8

[9] Vlaminck J Düsseldorf S Heres L Geldhof P. Serological examination of fattening pigs reveals associations between *Ascaris suum*, lung pathogens and technical performance parameters. Vet Parasitol. (2015) 210:151–8. 10.1016/j.vetpar.2015.04.012 25952722

[10] Hale OM Stewart TB Marti OG Wheat BE Mccormick WC. Influence of an experimental infection of nodular worms (*Oesophagostomum* spp.) on performance of pigs. J Anim Sci. (1981) 52:316–22. 10.2527/jas1981.522316x 7275858

[11] Stewart TB Gasbarre LC. The veterinary importance of nodular worms (*Olesophagostomum* spp). Parasitol Today. (1989) 5:209–13. 10.1016/0169-4758(89)90269-X 15463216

[12] Daugschies A Imarom S Ganter M Bollwahn W. Prevalence of *Eimeria* spp. in sows at piglet-producing farms in Germany. J Vet Med B Infect Dis Vet Public Health. (2004) 51:135–9. 10.1111/j.1439-0450.2004.00734.x 15107040

[13] Bangoura B Daugschies A. Eimeria. In: Florin-Christensen M, Schnittger L, editors. Parasitic Protozoa of Farm Animals and Pets. Cham: Springer International Publishing (2018). p. 55–101. 10.1007/978-3-319-70132-5_3

[14] Williams AR Krych L Fauzan Ahmad H Nejsum P Skovgaard K Nielsen DS. A polyphenol-enriched diet and *Ascaris suum* infection modulate mucosal immune responses and gut microbiota composition in pigs. PLoS ONE. (2017) 12:e0186546. 10.1371/journal.pone.0186546 29028844 PMC5640243

[15] Roepstorff A Bjørn H Nansen P. Resistance of *Oesophagostomum* spp. in pigs to pyrantel citrate. Vet Parasitol. (1987) 24:229–39. 10.1016/0304-4017(87)90044-6 2956755

[16] Gerwert S Failing K Bauer C. Prevalence of levamisole and benzimidazole resistance in *Oesophagostomum* populations of pig-breeding farms in North Rhine-Westphalia, Germany. Parasitol Res. (2002) 88:63–8. 10.1007/s004360100507 11822739

[17] Macrelli M Williamson S Mitchell S Pearson R Andrews L Morrison AA. First detection of ivermectin resistance in oesophagostomum dentatum in pigs. Vet Parasitol. (2019) 270:1–6. 10.1016/j.vetpar.2019.05.002 31213235

[18] Pettersson E Halvarsson P Sjölund M Grandi G Wallgren P Höglund J. First report on reduced efficacy of ivermectin on *Oesophagostomum* spp. on Swedish pig farms. Vet Parasitol Reg Stud Rep. (2021) 25:100598. 10.1016/j.vprsr.2021.100598 34474791

[19] Ihler CF. Anthelmintic resistance. An overview of the situation in the Nordic countries. Acta Veterinaria Scandinavica. (2010) 52:S24. 10.1186/1751-0147-52-S1-S24

[20] Armstrong SK Woodgate RG Gough S Heller J Sangster NC Hughes KJ. The efficacy of ivermectin, pyrantel and fenbendazole against *Parascaris equorum* infection in foals on farms in Australia. Vet Parasitol. (2014) 205:575–80. 10.1016/j.vetpar.2014.08.028 25224788

[21] Krücken J Fraundorfer K Mugisha JC Ramünke S Sifft KC Geus D. Reduced efficacy of albendazole against *Ascaris lumbricoides* in Rwandan schoolchildren. Int J Parasitol Drugs Drug Resist. (2017) 7:262–71. 10.1016/j.ijpddr.2017.06.001 28697451 PMC5503839

[22] Collins JB Jordan B Baldwin L Hebron C Paras K Vidyashankar AN. Resistance to fenbendazole in *Ascaridia dissimilis*, an important nematode parasite of turkeys. Poult Sci. (2019) 98:5412–5. 10.3382/ps/pez379 31328783

[23] Shalaby HA. Anthelmintics resistance; how to overcome it? Iran J Parasitol. (2013) 8:18–32. 23682256 PMC3655236

[24] Hoste H Torres-Acosta JF Sandoval-Castro CA Mueller-Harvey I Sotiraki S Louvandini H. Tannin containing legumes as a model for nutraceuticals against digestive parasites in livestock. Vet Parasitol. (2015) 212:5–17. 10.1016/j.vetpar.2015.06.026 26190131

[25] Peña-Espinoza M Thamsborg SM Desrues O Hansen TV Enemark HL. Anthelmintic effects of forage chicory (*Cichorium intybus*) against gastrointestinal nematode parasites in experimentally infected cattle. Parasitology. (2016) 143:1279–93. 10.1017/S0031182016000706 27173405 PMC4988272

[26] Štrbac F Krnjajić S Maurelli MP Stojanović D Simin N Orčić D. A potential anthelmintic phytopharmacological source of *Origanum vulgare* (L.) essential oil against gastrointestinal nematodes of sheep. Animals. (2022) 13:45. 10.3390/ani13010045 36611652 PMC9817997

[27] Van Krimpen MM Binnendijk GP Borgsteede FH Gaasenbeek CP. Anthelmintic effects of phytogenic feed additives in *Ascaris suum* inoculated pigs. Vet Parasitol. (2010) 168:269–77. 10.1016/j.vetpar.2009.11.004 19954891

[28] Băieş MH Cotutiu VD Spînu M Mathe A Cozma-Petru? A Bolboacǎ SD. *In vivo* assessment of the antiparasitic effects of *Allium sativum* L. and *Artemisia absinthium* L against gastrointestinal parasites in swine from low-input farms. BMC Vet Res. (2024) 20:126. 10.1186/s12917-024-03983-3 38561770 PMC10983701

[29] Bonde CS Bornancin L Lu Y Simonsen HT Martínez-Valladares M Peña-Espinoza M. Bio-guided fractionation and molecular networking reveal fatty acids to be principal anti-parasitic compounds in Nordic seaweeds. Front Pharmacol. (2021) 12:674520. 10.3389/fphar.2021.674520 34149425 PMC8206555

[30] Geisshirt HA Bonde CS Marcussen C Mejer H Williams AR. Development of *in vitro* assays with the canine hookworm uncinaria stenocephala and assessment of natural plant products for anti-parasitic activity. Pathogens. (2023) 12:536. 10.3390/pathogens12040536 37111422 PMC10144190

[31] Bonde CS Mejer H Myhill LJ Zhu L Jensen P Büdeyri Gökgöz N. Dietary seaweed (*Saccharina latissima*) supplementation in pigs induces localized immunomodulatory effects and minor gut microbiota changes during intestinal helminth infection. Sci Rep. (2023) 13:21931. 10.1038/s41598-023-49082-5 38081984 PMC10713666

[32] Rioux LE Turgeon SL Beaulieu M. Characterization of polysaccharides extracted from brown seaweeds. Carbohydr Polym. (2007) 69:530–7. 10.1016/j.carbpol.2007.01.009

[33] Gupta S Abu-Ghannam N. Bioactive potential and possible health effects of edible brown seaweeds. Trends Food Sci Technol. (2011) 22:315–26. 10.1016/j.tifs.2011.03.011

[34] Holdt SL Kraan S. Bioactive compounds in seaweed: functional food applications and legislation. J Appl Phycol. (2011) 23:543–97. 10.1007/s10811-010-9632-5

[35] You L Gong Y Li L Hu X Brennan C Kulikouskaya V. Beneficial effects of three brown seaweed polysaccharides on gut microbiota and their structural characteristics: an overview. Int J Food Sci Technol. (2020) 55:1199–206. 10.1111/ijfs.14408

[36] Ribeiro DM Leclercq CC Charton SB Costa MM Carvalho DFP Cocco E. Enhanced ileum function in weaned piglets via *Laminaria digitata* and alginate lyase dietary inclusion: a combined proteomics and metabolomics analysis. J Proteomics. (2023) 289:105013. 10.1016/j.jprot.2023.105013 37775079

[37] Petkevicius S Bach Knudsen KE Murrell KD Wachmann H. The effect of inulin and sugar beet fibre on *Oesophagostomum dentatum* infection in pigs. Parasitology. (2003) 127:61–8. 10.1017/S0031182003003251 12885189

[38] Peachey LE Jenkins TP Cantacessi C. This gut ain't big enough for both of us. Or is it? Helminth–microbiota interactions in veterinary species. Trends Parasitol. (2017) 33:619–32. 10.1016/j.pt.2017.04.004 28506779

[39] Veronico P Melillo MT. Marine organisms for the sustainable management of plant parasitic nematodes. Plants. (2021) 10:369. 10.3390/plants10020369 33672987 PMC7918792

[40] Hui Y Tamez-Hidalgo P Cieplak T Satessa GD Kot W Kjærulff S. Supplementation of a lacto-fermented rapeseed-seaweed blend promotes gut microbial- and gut immune-modulation in weaner piglets. J Anim Sci Biotechnol. (2021) 12:85. 10.1186/s40104-021-00601-2 34281627 PMC8290543

[41] Umu ÖCO Mydland LT Øverland M Press CM Sørum H. Rapeseed-based diet modulates the imputed functions of gut microbiome in growing-finishing pigs. Sci Rep. (2020) 10:9372. 10.1038/s41598-020-66364-4 32523033 PMC7287078

[42] Skugor A Kjos NP Sundaram AYM Mydland LT Ånestad R Tauson AH. Effects of long-term feeding of rapeseed meal on skeletal muscle transcriptome, production efficiency and meat quality traits in Norwegian Landrace growing-finishing pigs. PLoS ONE. (2019) 14:e0220441. 10.1371/journal.pone.0220441 31390356 PMC6685631

[43] Øverland M Mydland LT Skrede A. Marine macroalgae are sources of protein and bioactive compounds in feed for monogastric animals. J Sci Food Agric. (2019) 99:13–24. 10.1002/jsfa.9143 29797494 PMC6585948

[44] Wang Y Liu J Wei F Liu X Yi C Zhang Y. Improvement of the nutritional value, sensory properties and bioavailability of rapeseed meal fermented with mixed microorganisms. LWT. (2019) 112:108238. 10.1016/j.lwt.2019.06.005

[45] Satessa GD Tamez-Hidalgo P Hui Y Cieplak T Krych L Kjærulff S. Impact of Dietary supplementation of lactic acid bacteria fermented rapeseed with or without macroalgae on performance and health of piglets following omission of medicinal zinc from weaner diets. Animals. (2020) 10:137. 10.3390/ani10010137 31952154 PMC7023219

[46] Xu B Li Z Wang C Fu J Zhang Y Wang Y. Effects of fermented feed supplementation on pig growth performance: a meta-analysis. Anim Feed Sci Technol. (2020) 259:114315. 10.1016/j.anifeedsci.2019.114315

[47] Satessa GD Tamez-Hidalgo P Kjærulff S Vargas-Bello-Pérez E Dhakal R Nielsen MO. Effects of increasing doses of lactobacillus pre-fermented rapeseed product with or without inclusion of macroalgae product on weaner piglet performance and intestinal development. Animals. (2020) 10:559. 10.3390/ani10040559 32230825 PMC7222423

[48] Petersen HH Takeuchi-Storm N Enemark HL Nielsen ST Larsen G Chriél M. Surveillance of important bacterial and parasitic infections in Danish wild boars (*Sus scrofa*). Acta Vet Scand. (2020) 62:41. 10.1186/s13028-020-00539-x 32746868 PMC7398403

[49] Vlaminck J Nejsum P Vangroenweghe F Thamsborg SM Vercruysse J Geldhof P. Evaluation of a serodiagnostic test using *Ascaris suum* haemoglobin for the detection of roundworm infections in pig populations. Vet Parasitol. (2012) 189:267–73. 10.1016/j.vetpar.2012.04.024 22560331

[50] De Coster W D'hert S Schultz DT Cruts M Van Broeckhoven C. NanoPack: visualizing and processing long-read sequencing data. Bioinformatics. (2018) 34:2666–9. 10.1093/bioinformatics/bty149 29547981 PMC6061794

[51] Caporaso JG Kuczynski J Stombaugh J Bittinger K Bushman FD Costello EK. QIIME allows analysis of high-throughput community sequencing data. Nat Methods. (2010) 7:335–6. 10.1038/nmeth.f.303 20383131 PMC3156573

[52] McDonald D Price MN Goodrich J Nawrocki EP Desantis TZ Probst A. An improved Greengenes taxonomy with explicit ranks for ecological and evolutionary analyses of bacteria and archaea. ISME J. (2012) 6:610–8. 10.1038/ismej.2011.139 22134646 PMC3280142

[53] Boes J Nansen P Stephenson LS. False-positive *Ascaris suum* egg counts in pigs. Int J Parasitol. (1997) 27:833–8. 10.1016/S0020-7519(97)00054-4 9279587

[54] Bolyen E Rideout JR Dillon MR Bokulich NA Abnet CC Al-Ghalith GA. Reproducible, interactive, scalable and extensible microbiome data science using QIIME 2. Nat Biotechnol. (2019) 37:852–7. 10.1038/s41587-019-0209-9 31341288 PMC7015180

[55] R Core Team. R: A Language and Environment for Statistical Computing. Vienna: R Foundation for Statistical Computing (2020).

[56] McMurdie PJ Holmes S. phyloseq: an R package for reproducible interactive analysis and graphics of microbiome census data. PLoS ONE. (2013) 8:e61217. 10.1371/journal.pone.0061217 23630581 PMC3632530

[57] Wickham H. Welcome to the tidyverse. J Open Source Softw. (2019) 4:1686. 10.21105/joss.01686

[58] Kassambara A. “*ggplot2” Based Publication Ready Plots*. (2020). Available online at: https://CRAN.R-project.org/package=ggpubr

[59] Wickham H. Reshaping data with the reshape package. J Stat Softw. (2007) 21:1–20. 10.18637/jss.v021.i12

[60] Dawson C. ggprism: A ‘ggplot2' Extension Inspired by ‘GraphPad Prism'. R package version 1.0.5 (2024).

[61] Love MI Huber W Anders S. Moderated estimation of fold change and dispersion for RNA-seq data with DESeq2. Genome Biol. (2014) 15:550. 10.1186/s13059-014-0550-8 25516281 PMC4302049

[62] Kolde R. Pretty Heatmaps. (2019). Available online at: https://rdrr.io/cran/pheatmap/

[63] Neuwirth E. ColorBrewer Palettes. (2014). Available online at: https://CRAN.R-project.org/package=RColorBrewer

[64] Herve M. RVAideMemoire: Testing and Plotting Procedures for Biostatistics. (2021). Available online at: https://CRAN.R-project.org/package=RVAideMemoire

[65] Leonard SG Sweeney T Bahar B Lynch BP O'doherty JV. Effects of dietary seaweed extract supplementation in sows and post-weaned pigs on performance, intestinal morphology, intestinal microflora and immune status. Br J Nutr. (2011) 106:688–99. 10.1017/S0007114511000997 21736851

[66] Avery JL. The effect of moderately low temperatures on the sporulation of oocysts of two species of swine coccidia. J Parasitol. (1942) 28:27–8.

[67] Lee MB Lee EH. Coccidial contamination of raspberries: mock contamination with Eimeria acervulina as a model for decontamination treatment studies. J Food Prot. (2001) 64:1854–7. 10.4315/0362-028X-64.11.1854 11726175

[68] Lassen B Seppa-Lassila L. Recovery and sporulation of bovine *Eimeria* oocysts after exposure to sub-zero temperature. Vet Zoot. (2014) 66:35–9.

[69] Cram GB. The influence of low temperatures and of disinfectants on the eggs of *Ascaris lumbricoides*. J Agric Res. (1924) 27:167–75.

[70] Roepstorff A Eriksen L Slotved HC Nansen P. Experimental *Ascaris suum* infection in the pig: worm population kinetics following single inoculations with three doses of infective eggs. Parasitology. (1997) 115(Pt 4):443–52. 10.1017/S0031182097001480 9364572

[71] Eriksen L Nansen P Roepstorff A Lind P Nilsson O. Response to repeated inoculations with *Ascaris suum* eggs in pigs during the fattening period. I. Studies on worm population kinetics. Parasitol Res. (1992) 78:241–6. 10.1007/BF00931733 1534170

[72] Roepstorff A. Natural *Ascaris suum* infections in swine diagnosed by coprological and serological (ELISA) methods. Parasitol Res. (1998) 84:537–43. 10.1007/s004360050444 9694368

[73] Bøgh HO Eriksen L Lawson LG Lind P. Evaluation of an enzyme-linked immunosorbent assay and a histamine release test system for the detection of pigs naturally infected with *Ascaris suum*. Prev Vet Med. (1994) 21:201–14. 10.1016/0167-5877(94)90019-1

[74] Petkevicius S Thomsen LE Bach Knudsen KE Murrell KD Roepstorff A Boes J. The effect of inulin on new and on patent infections of *Trichuris suis* in growing pigs. Parasitology. (2007) 134:121–7. 10.1017/S0031182006000977 17032472

[75] Petkevicius S Knudsen KE Nansen P Murrell KD. The effect of dietary carbohydrates with different digestibility on the populations of *Oesophagostomum dentatum* in the intestinal tract of pigs. Parasitology. (2001) 123:315–24. 10.1017/S0031182001008472 11578096

[76] Devillé C Damas J Forget P Dandrifosse G Peulen O. Laminarin in the dietary fibre concept. J Sci Food Agric. (2004) 84:1030–8. 10.1002/jsfa.1754

[77] Wang Y Han F Hu B Li J Yu W. *In vivo* prebiotic properties of alginate oligosaccharides prepared through enzymatic hydrolysis of alginate. Nutr Res. (2006) 26:597–603. 10.1016/j.nutres.2006.09.015

[78] Wang X Huang M Yang F Sun H Zhou X Guo Y. Rapeseed polysaccharides as prebiotics on growth and acidifying activity of probiotics *in vitro*. Carbohydr Polym. (2015) 125:232–40. 10.1016/j.carbpol.2015.02.040 25857979

[79] Wang X Tsai T Deng F Wei X Chai J Knapp J. Longitudinal investigation of the swine gut microbiome from birth to market reveals stage and growth performance associated bacteria. Microbiome. (2019) 7:109. 10.1186/s40168-019-0721-7 31362781 PMC6664762

[80] Gnoth S Jenzsch M Simutis R Lübbert A. Process analytical technology (PAT): batch-to-batch reproducibility of fermentation processes by robust process operational design and control. J Biotechnol. (2007) 132:180–6. 10.1016/j.jbiotec.2007.03.020 17559961

[81] Vigors S O'doherty JV Rattigan R Mcdonnell MJ Rajauria G Sweeney T. Effect of a laminarin rich macroalgal extract on the caecal and colonic microbiota in the post-weaned pig. Mar Drugs. (2020) 18:157. 10.3390/md18030157 32168972 PMC7143854

[82] Zhao J Tian H Kong X Dang D Liu K Su C. Microbiomic and Metabolomic Insights into the mechanisms of alfalfa polysaccharides and seaweed polysaccharides in alleviating diarrhea in pre-weaning holstein calves. Animals. (2025) 15:485. 10.3390/ani15040485 40002967 PMC11851682

[83] Zhou M Zeng Y Zhou W Zhu H Xing Y Dong X. Supplementation of *Laminaria japonica* powder influence ruminal microbiota without affecting ruminal fermentation in bulls. Pak Vet J. (2024) 44:1255–62. 10.29261/pakvetj/2024.265

[84] Chen C Fang S Wei H He M Fu H Xiong X. Prevotella copri increases fat accumulation in pigs fed with formula diets. Microbiome. (2021) 9:175. 10.1186/s40168-021-01110-0 34419147 PMC8380364

[85] Naczk M Amarowicz R Pink D Shahidi F. Insoluble condensed tannins of canola/rapeseed. J Agric Food Chem. (2000) 48:1758–62. 10.1021/jf9908401 10820091

[86] Athanasiadou S Githiori J Kyriazakis I. Medicinal plants for helminth parasite control: facts and fiction. Animal. (2007) 1:1392–400. 10.1017/S1751731107000730 22444894

[87] Forgie AJ Fouhse JM Willing BP. Diet-microbe-host interactions that affect gut mucosal integrity and infection resistance. Front Immunol. (2019) 10:1802. 10.3389/fimmu.2019.01802 31447837 PMC6691341

[88] Lipsa FD Snowdon R Friedt W. Quantitative genetic analysis of condensed tannins in oilseed rape meal. Euphytica. (2012) 184:195–205. 10.1007/s10681-011-0546-3

[89] Ribeiro DM Martins CF Costa M Coelho D Pestana J Alfaia C. Quality traits and nutritional value of pork and poultry meat from animals fed with seaweeds. Foods. (2021) 10:2961. 10.3390/foods10122961 34945510 PMC8701104

[90] Lindberg JE. Fiber effects in nutrition and gut health in pigs. J Anim Sci Biotechnol. (2014) 5:15. 10.1186/2049-1891-5-15 24580966 PMC3975931

[91] Skitsko PJ Bowland JP. Performance of gilts and barrows from three breeding groups marketed at three liveweights when offered diets containing two levels of digestible energy for a limited period per day. Can J Anim Sci. (1970) 50:161–70. 10.4141/cjas70-020

[92] Garitano I Liébana C De Vargas EF Olivares Á Daza A. Effect of gender on growth performance, carcass characteristics, meat and fat composition of pigs slaughtered at 125 kg of live weight destined to teruel (Spain) ham production. Ital J Anim Sci. (2013) 12:e16. 10.5424/sjar/2014123-4693

[93] Jaturashita S Kamopas S Suppadit T Khiaosa-Ard R Kreuzer M. The effect of gender of finishing pigs slaughtered at 110 kilograms on performance, and carcass and meat quality. ScienceAsia. (2006) 32:297–305. 10.2306/scienceasia1513-1874.2006.32.297

[94] Stygar AH Dolecheck KA Kristensen AR. Analyses of body weight patterns in growing pigs: a new view on body weight in pigs for frequent monitoring. Animal. (2018) 12:295–302. 10.1017/S1751731117001690 28735585

[95] Nunn CL Altizer S Jones KE Sechrest W. Comparative tests of parasite species richness in primates. Am Nat. (2003) 162:597–614. 10.1086/378721 14618538

[96] Barger IA. Influence of sex and reproductive status on susceptibility of ruminants to nematode parasitism. Int J Parasitol. (1993) 23:463–9. 10.1016/0020-7519(93)90034-V 8354597

