# Supplementary material for: Dietary supplementation with fermented rapeseed and seaweed modulates parasite infections and gut microbiota in outdoor pigs
Source: Front Vet Sci. 2025 Jun 19;12:1565686. doi: 10.3389/fvets.2025.1565686 (PMC12223427; doi:10.3389/fvets.2025.1565686)
Supplement: Supplementary Table 1 — UMI containing multiple forward and reverse primers used in this study. [file Table_1.pdf]

Supplementary Table 1: UMI containing multiple forward and reverse primers used in this study.

Primers	Primer Sequence
UMI_338Fa	5’- GTCTCGTGGGCTCGG- NNNNNNNNNNNNNNN - ACWCCTACGGGWGGCAGCAG-3’
UMI_338Fb	5’- GTCTCGTGGGCTCGG- NNNNNNNNNNNNNNN - GACTCCTACGGGAGGCWGCAG-3’
UMI_27Fa	5’- GTCTCGTGGGCTCGG- NNNNNNNNNNNNNNN - AGAGTTTGATYMTGGCTYAG-3’
UMI_27Fb	5’- GTCTCGTGGGCTCGG- NNNNNNNNNNNNNNN - AGGGTTCGATTCTGGCTCAG-3’
UMI_1540R	5’- GTCTCGTGGGCTCGG- NNNNNNNNNNNNNNN - TACGGYTACCTTGTTACGACT-3’
UMI_1391R	5’- GTCTCGTGGGCTCGG- NNNNNNNNNNNNNNN - GACGGGCGGTGTGTRCA-3’
