# Supplementary material for: Dietary supplementation with fermented rapeseed and seaweed modulates parasite infections and gut microbiota in outdoor pigs
Source: Front Vet Sci. 2025 Jun 19;12:1565686. doi: 10.3389/fvets.2025.1565686 (PMC12223427; doi:10.3389/fvets.2025.1565686)
Supplement: Supplementary Table 2 — P-values for the final model of fecal egg count (FEC), based on logarithmic transformation for weeks 9 and 12 or 11. Includes Sub-study 1 (SUB1) and combined Sub-studies 2–4 (SUB2–4). P-values are from the final model, whereas “Excl.” refers to factors excluded from the final model (>0.15). [file Table_2.pdf]

Supplementary Table 2: P-values for the final model of faecal egg count (FEC), based on logarithmic transformation for week 9 and 12 or 11. Includes Sub-study 1 (SUB1) and combined Sub-studies 2-4 (SUB2-4). P-values are from the final model whereas, 'Excl.' refers to factors excluded from the final model (>0.15).

**Week**	**SUB**	DWG	Diet	Sex	Sub-study
**9**	**1**	Excl.	0.90	Excl.	n.a.
	**2-4**	0.08	0.08	0.01	0.61
**12**	**1**	0.10	0.25	Excl.	n.a.
**11**	**2-4**	Excl.	0.10	0.02	0.72
