# Supplementary material for: Dietary supplementation with fermented rapeseed and seaweed modulates parasite infections and gut microbiota in outdoor pigs
Source: Front Vet Sci. 2025 Jun 19;12:1565686. doi: 10.3389/fvets.2025.1565686 (PMC12223427; doi:10.3389/fvets.2025.1565686)
Supplement: Supplementary Figure 1 — Mean (+ SD) optical density ratio (ODr) as measurement of serum IgG antibodies against adult (As-Hb) and larvae (L3 lung) Ascaris suum in pigs from for (A) sub-study 1, group C1 and S1; (B) Response when divided into FEC positive or FEC negative based on FEC >200 for sub-study 1; and (C) sub-study 2, group C2 and S2; (D) Response when divided into FEC positive or FEC negative based on EPG >200 for sub-study 2 (****P <0.0001). The stippled line represents the cut-off value for As-Hb (0.50) and L3 lung test (0.25). [file Image_1.pdf]

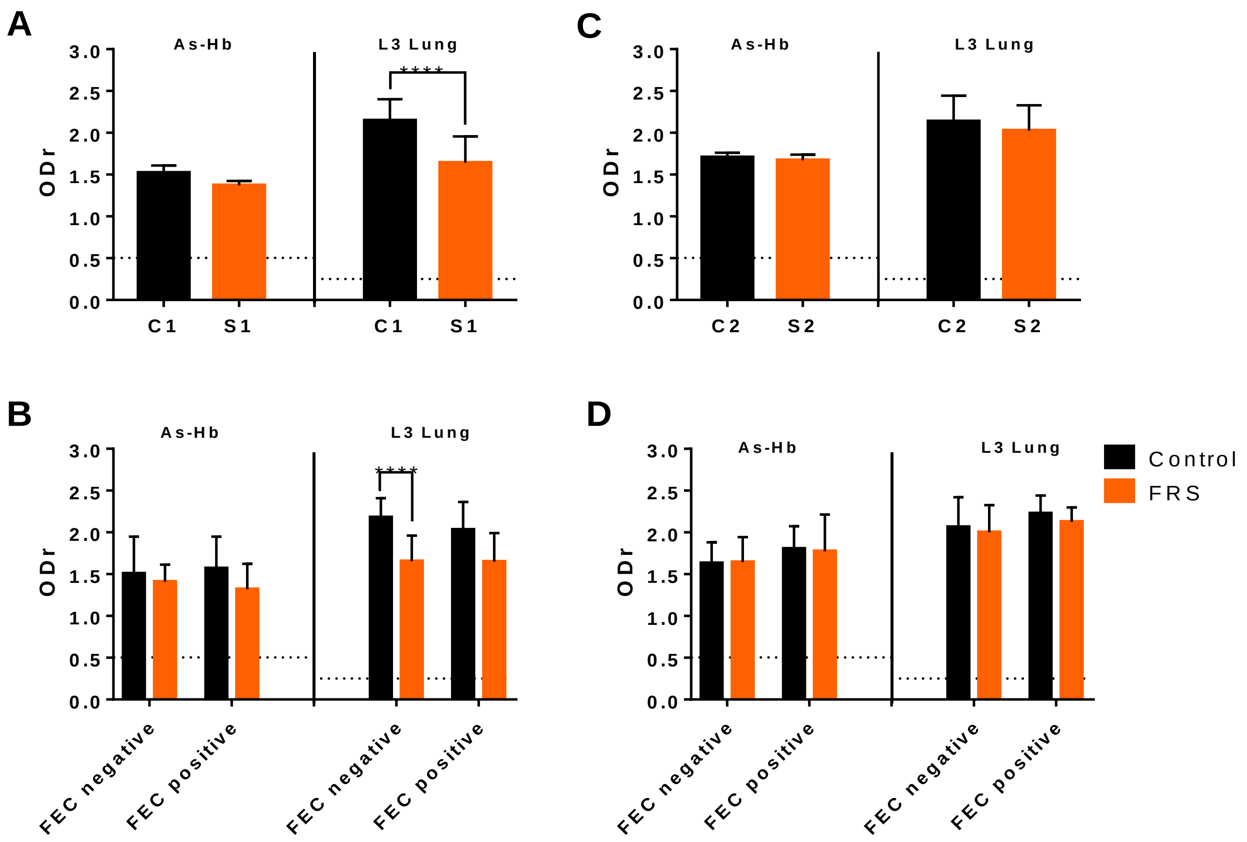

Supplementary Figure 1: Mean (+ SD) optical density ratio (ODr) as measurement of serum IgG antibodies against adult (As-Hb) and larvae (L3 lung) *Ascaris suum* in pigs from for; (**A**) sub-study 1, group C1 and S1; (**B**) Response when divided into FEC positive or FEC negative based on FEC>200 for sub-study 1; and (**C**) sub-study 2, group C2 and S2; (**D**) Response when divided into FEC positive or FEC negative based on EPG>200 for sub-study 2. (****P<0.0001). Stippled line represents cut-off value for As-Hb (0.50) and L3 lung test (0.25).
