# Supplementary material for: Dietary supplementation with fermented rapeseed and seaweed modulates parasite infections and gut microbiota in outdoor pigs
Source: Front Vet Sci. 2025 Jun 19;12:1565686. doi: 10.3389/fvets.2025.1565686 (PMC12223427; doi:10.3389/fvets.2025.1565686)
Supplement: Supplementary Figure 2 — Heatmap of bacteria found to be significantly differently abundant by DESeq2 analysis for SUB1 at week 0. The heatmap shows the comparison of control-fed (C) and FRS-fed (S) groups. Significantly different bacteria (Adjusted P-value < 0.05) at the species level are demonstrated in the heatmap. [file Image_2.pdf]

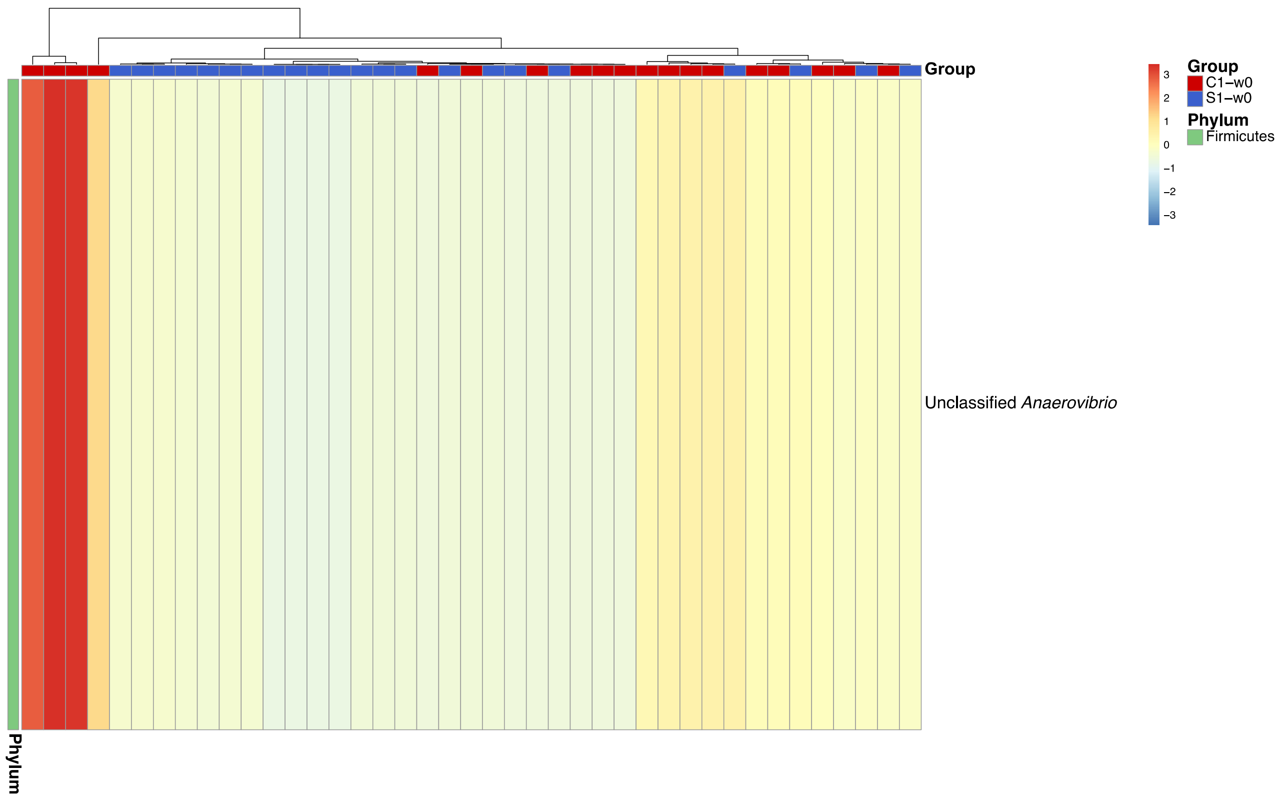

Supplementary Figure 2: Heatmap of bacteria found to being significantly differently abundant by DESeq2 analysis for SUB1 at week 0. Heatmap shows the comparison of control-fed (C) and FRS-fed (S) groups. Significantly different bacteria (Adjusted p-value<0.05) at species level are demonstrated in the heatmap.
